# Exploring Prognostic Immune Microenvironment-Related Genes in Head and Neck Squamous Cell Carcinoma from the TCGA Database

**DOI:** 10.7150/jca.89581

**Published:** 2024-01-01

**Authors:** Shuangjiang Li, Yiyu Zeng, Liming He, Xiaoyan Xie

**Affiliations:** 1 Department of Stomatology, Changsha Stomatological Hospital, Changsha, P. R. China.; 2 Department of Stomatology, The Second Xiangya Hospital, Central South University, Changsha, P. R. China.

**Keywords:** neck squamous cell carcinoma, tumor microenvironment, ESTIMATE, stromal score, immune score

## Abstract

**Purpose:** Head and neck squamous cell carcinoma (HNSCC) has a high rate of local and distant metastases. In tumor tissues, the interaction between tumor cells and the tumor microenvironment (TME) is closely related to cancer development and prognosis. Therefore, screening for TME-related genes in HNSCC is crucial for understanding metastatic patterns.

**Methods:** Our research relied mainly on a novel algorithm called Estimation of STromal and Immune cells in MAlignant Tumors using Expression data (ESTIMATE). Fragments Per Kilobase of exon model per Million mapped fragments (FPKM) data and HNSCC clinical data were obtained from the TCGA database, and the purity of HNSCC tissue and the features of stromal and immune cell infiltration were determined. Furthermore, differentially expressed genes (DEGs) were screened based on immune, stromal, and ESTIMATE scores, and their protein-protein interaction (PPI) networks and ClueGO functions were evaluated. Finally, the expression profiles of DEGs related to immunity in HNSCC were determined. Differential gene expression was verified in the highly invasive oral cancer cell lines (SCC-25, CAL-27, and FaDu) and oral cancer tissues.

**Results:** Our analysis found that both the immune and ESTIMATE scores were significantly associated with the prognosis of HNSCC. Moreover, cross-validation using the Venn algorithm revealed that 433 genes were significantly upregulated, and 394 genes were significantly downregulated. All DEGs were associated with both ESTIMATE and immune scores. The enrichment of cytokine-cytokine receptor interactions and chemokine signaling pathways was observed using pathway enrichment analyses. We initially screened 25 genes after analyzing the key sub-networks of the PPI network. Survival analysis revealed the significance of CCR4, CXCR3, P2RY14, CCR2, CCR8, and CCL19 in relation to survival and their association with immune infiltration-related metastasis in HNSCC.

**Conclusions:** The expression profiles of relevant TME-related genes were screened following stromal and immune cell scoring using ESTIMATE, and DEGs associated with survival were identified. These TME-related gene markers offer valuable utility as both prognostic indicators and markers denoting metastatic traits in HNSCC.

## Introduction

Head and neck squamous cell carcinoma (HNSCC) arises from the squamous cell epithelium and comprises heterogeneous tumors of the head and neck, including the oral cavity, oropharynx, larynx, and hypopharynx 1. Although the survival rate of patients with HNSCC has improved significantly in recent years due to improvements in cancer treatment modalities, the incidence of local and distant metastases in patients with advanced HNSCC who receive treatment is still as high as 40% and 30%, respectively 2. Cancer remains a major global health challenge, exacting a growing toll on societies worldwide 3. It is important to identify factors that affect treatment prognosis in patients with HNSCC as this could lead to the development of strategies to improve treatment and prognosis in the future.

The tumor microenvironment (TME) plays an important role in tissue homeostasis and tumor development. In tumor tissues, the interaction between cells and the associated stroma is closely related to the development and prognosis of cancer 4. Tumor cells have a complex regulatory relationship with their microenvironmental composition and stromal cells, which play a role in their response to extrinsic and intrinsic factors, including environmental factors and proto-oncogene expression 4,5. In fact, one of the direct causes of tumorigenesis is the dysregulation of the TME and subsequent chronic inflammation in tissues 6. Furthermore, the TME is critical for tumor development, and local tumor immune responses are often coordinated across tissues and involve a wide variety of cells 7. In a clinical context, antibodies targeting PD-1/PD-L1 have demonstrated the ability to reactivate "depleted" T cells within the TME, exhibiting anti-tumor properties across a wide spectrum of cancers, including melanoma and lymphoma 8. Notably, a wide variety of immune cells are involved in cancer development 9,10. Macrophage consumption in non-small cell lung cancer reduces the number and phenotype of regulatory T cells and promotes the accumulation of CD8+T cells, thus reducing tumor invasion and growth 11. Therefore, a comprehensive analysis of HNSCC tissue, its microenvironment, and related mechanistic cells would be useful for screening new target genes for HNSCC treatment and improving prognosis.

Most current studies on cancer-related gene expression profiles are based on the direct analysis of tumor tissue 12,13. In the case of metastatic tumors driven by crosstalk between cancer cells and the TME, tumor-associated immune cells elicit tumor cell immune escape by secreting cytokines, chemokines, and growth factors into the TME, leading to tumor infiltration in other tissues and organs tumor infiltration 14. Therefore, analyzing the gene expression profiles of TME-associated tissues is critical for understanding the molecular pathways involved in tumor metastasis. There is some evidence that sample heterogeneity in TME gene expression analysis is influenced by the infiltration of normal cells into the TME, and that the inclusion of infiltrating tumor-associated normal cells in the genomic expression analysis of tumor samples has an impact on the results of the analysis 15. Accordingly, the calculation of immune cell purity has attracted much attention in the bioinformatics analysis of tumor samples and has been applied in this study.

Most previous studies utilizing gene bioinformatics analysis have focused on differences in the transcriptome characteristics of different types of cells or specific cell expression profiles 16-18. Instead, in this study, we determined the purity of the tumor tissue and the specific features of stromal and immune cell infiltration using a novel algorithm called Estimation of STromal and Immune cells in Malignant Tumours using Expression data (ESTIMATE) 19. The ESTIMATE algorithm divides tumor samples into stromal scores (stroma in tumor tissue), immune scores (infiltration of immune cells in tumor tissue), and ESTIMATE scores (tumor purity). Using the TCGA database, we introduced a new hierarchical model of potential gene features based on matrix and immune scores. Stromal and immune cells in the tumor tissue were subdivided to assess possible immune-related gene differences. Our aim was to use the unique characteristics of the transcriptional profiles of HNSCC cancer samples to infer the nature of cells in the TME using a bioinformatics approach, as well as determining the association of these cells with survival and prognosis. Additionally, we determined the differential expression profile of immune-related HNSCC genes. Through survival analysis of these genes, we were able to identify potential treatment targets and prognostic indicators for future research.

## Methods

### Data download and processing

Fragments Per Kilobase of exon model per million mapped fragments (FPKM) data and clinical data for HNSCC were downloaded from the TCGA website (https://portal.gdc.cancer.gov/). We screened samples based on clinical information. Based on the inclusion criteria, only primary tumor tissue samples from patients with survival times greater than one month and less than five years were included. The samples for which survival information was unavailable were excluded. After the data were screened according to the above criteria, 439 HNSCC samples were included in the subsequent analysis. To facilitate subsequent analysis, we compared the FPKM data of HNSCC with genetic information sourced from the HGNC database (https://www.genenames.org/). We converted Ensembl IDs into gene symbols and EntrezIDs.

### Application of ESTIMATE for immune purity calculation

We extracted expression matrices for the samples included in the analysis and used the ESTIMATE R package to calculate the immune purity based on the expression matrices. Single-sample gene set enrichment analysis (ssGSEA) was used to calculate the stromal and immune scores of each sample, and the ESTIMATE score was determined by combining these two scores. Using the ESTIMATE R package following Yoshihara's paper, we first unified the gene identifiers of the input data against the common genes using the filterCommonGenes function and then calculated stromal, immune, and ESTIMATE scores by applying the estimateScore function, which is based on the ssGSEA algorithm.

### Tumor stage and survival according to the calculated scores

The stromal score (which captures the presence of stroma in tumor tissue), immune score (which represents the infiltration of immune cells in tumor tissue), and ESTIMATE score (which infers tumor purity and is equal in number to the stromal and immune scores) were plotted for different tumor stages. Boxplots were drawn to view the distribution of scores for different tumor stages. Survival analysis was performed to determine whether these scores were significantly associated with HNSCC prognosis. In the survival analysis, we first used the survival cutpoint function provided by the survminer R package to get the optimal cutpoint for each score. We used the optimal cutoff to group the scores as “high” if they were more than the optimal cutoff, and “low” if they were lower than the optimal cutoff. Subsequently, a survival analysis of the high- and low-scoring groups was performed.

### Analysis of differential gene expression

High- and low-scoring groups were assigned based on the optimal cutoff values of the three scores, as described earlier, and differential gene expression between the high- and low-scoring groups was evaluated. We downloaded the count data from the TCGA database for the samples included in our study and analyzed the differentially expressed genes (DEGs) using the Edge R package. Volcano plots were generated using the ggplot2 package in R. Differential analysis was based on the expression matrix of the screened samples. After the differential gene annotation of the expression matrix, we extracted protein-coding genes and lncRNAs from the expression matrix according to the human genome analysis published in the HGNC database. Analysis was performed using the Edge R package in R, and the thresholds for screening DEGs were |logFC| > 1 and P < 0.01.

### Cross-validation and functional enrichment of differentially expressed genes

Two sets of DEGs were identified based on immune and stromal scores. We then used the Venn algorithm to cross-validate the two sets of DEGs. Genes that were significantly upregulated based on both scores and those that were significantly downregulated based on both scores were regarded as the final set of significantly upregulated and downregulated genes, respectively. Next, we performed gene ontology (GO) and Kyoto Encyclopedia of Genes and Genomes (KEGG) pathway enrichment analyses of the DEGs using the clusterProfiler package in R and used bar charts and bubble charts to depict the enrichment results for the top 10 visualizations (verified using the ClueGO plugin in Cytoscape).

### Protein-protein interaction networks (PPI) analysis and ClueGO functional analysis

Using the significantly upregulated genes as the main analysis objects, we used the STRING database to perform PPI analysis of the significantly upregulated genes (the default analysis parameter was an interaction combined score > 0.7). Functional analyses were performed using the ClueGO plugin.

### Analysis of key sub-networks of the PPI network

We used Cytoscape's MCODE plugin to perform key sub-network analysis on the resulting PPI network using the following analysis parameters: MCODE score ≥ 5, degree cutoff = 2, node score cutoff = 0.2, max depth = 100, and k-score = 2. In our preliminary screening, we identified key genes within the sub-network boasting the highest score.

### Survival analysis and multivariate regression analysis

Survival analysis was performed on genes in the key sub-network (median value of cutoff, >cutoff as high, and <cutoff as low, followed by survival analysis of previous grouping results based on immune and stromal scores). Multivariate regression analysis was performed to assess the expression levels of key genes, stromal score, immune score, pathologic _T, pathologic _N, pathologic _M, and clinical stage as prognostic indicators (using the survivorship and survivor packages). The genes that showed the most significant association with cancer prognosis were identified (stromal score and immune score had no impact on survival; the previous analysis used the optimal cutoff point, so there was a significant difference).

### Cell culture

We purchased the highly invasive oral cancer cell lines SCC-25 (HTX1938), CAL-27 (HTX2447), and FaDu (HTX1735) and the less invasive cell line HSC-2 (HTX2346) from Otwo Biotech (Shenzhen, China). Cells were cultured in DMEM supplemented with 10% fetal bovine serum (FBS), 100 IU/mL penicillin, and 100 IU/mL streptomycin.

### Real-time quantitative PCR (qRT-PCR) for the detection of gene expression

Following the protocol outlined in a previous study 20, total RNA was extracted from the cell lines using TRIzol reagent (15596-026, Invitrogen, USA). Total RNA (1 μg) from each sample was reverse-transcribed into cDNA using a One-Step RT-PCR kit (RR036B, TaKaRa, Japan). For real-time PCR, we employed the One-Step TB Green™ PrimeScript™ RT-PCR Kit II (SYBR Green) (RR086B, TaKaRa, Japan), and utilized an ABI Step One Plus Real-time PCR system (USA) according to the manufacturer's instructions. GAPDH was used as a housekeeping gene, and the relative expression levels of the genes were calculated using the 2-(ΔCt) method. The primers were synthesized by KeyGEN Biosynthesis (3'-5'):

CCR4 (F-CTGTGGTGGTTCTGGTCCTGTTC; R-AGATCCGAGATGGCAAGGTTGAG),

CXCR3 (F-CCTTCCTGCTCCACCTAGCTGTAG; R-GCTCCTGCGTAGAAGTTGATGTTGA),

P2RY14 (F-GGTCTCTGAAACGTGCTCTTCTAC; R-TTGCTGTAACTCACTGACTGGATGA),

CCR2 (F-CCTGAGTC) R-GAGTAGAGCGGAGGCAGGAGTT),

CCR8 (F-GGTTGGTGCTCATTGTGGTCATTG; R-GCTGTTGGCTTATGCTACATCCATC),

CCL19 (F-GCCTGCTGGTTCTCTGGACTTC; R-AGGGATGGGTTTCTGGGTCACA),

and GAPDH (F-AGATCATCAGCAATGCCTCCT; R-TGAGTCCTTCCACGATACCAA).

### Western blot analysis

Total protein in the cells was extracted using RIPA lysis buffer (89901, Invitrogen) and quantified using a BCA kit (23225, Invitrogen). Total protein (20 µg) was separated using 10% SDS-PAGE and transferred to a polyvinylidene fluoride membrane (Millipore, Bedford, USA). The membrane was blocked with 5% nonfat milk and incubated with primary antibodies overnight at 4°C. The membranes were then incubated with the corresponding secondary antibodies for 2 h at room temperature. The protein bands were detected using the ECL detection kit (Millipore). The signal intensity was analyzed using ImageJ software. The following primary antibodies were used: anti-CCR4 (1:1,000, ab254376, Abcam, Cambridge, UK), anti-CXCR3 (1:1,000, ab288437, Abcam, Cambridge, UK), anti-P2RY14 (1:1,000, ab136264, Abcam, Cambridge, UK), anti-CCR2 (1:1,500, #12199, Cell Signaling Technology, Massachusetts, USA), anti-CCR8 (1:1,000, ab32399, Abcam, Cambridge, UK), and anti-CCL19 (1:1,000, ab192877, Abcam, Cambridge, UK).

### Clinical patient sample collection

All 10 pairs of tongue or oral cancers with lymph or distant metastases and tumor tissues without metastasis were obtained from the Department of Stomatology, the Second Xiangya Hospital. Patients with metastases exhibited lymphatic or distant metastatic tongue or oral cancers, whereas non-metastatic patients remained free from metastasis (Table 1). All patients underwent radiotherapy, chemotherapy, and immunotherapy and did not have any other serious diseases. All studies were conducted with written patient consent, approved by the Ethics Committee of the Second Xiangya Hospital, Central South University.

### Immunohistochemistry

HNSCC tissues were paraffin-embedded and sectioned, as previously reported in the literature 21, Briefly, the slices were dewaxed with different concentrations of xylene and rehydrated with ethanol. Next, the slices were incubated in 3% hydrogen peroxide solution containing methanol for 15 min to block endogenous peroxidase activity. The slices were heated in 100 mmol/L sodium citrate buffer (pH 6.0) for 10 min to recover the antigen and then left at room temperature for 20 min. After washing with PBS, the slices were incubated with primary antibody. Following color development, we applied cover slips to the sections and observed them in five random fields at 200× magnification under a light microscope. Positively labeled cell percentages were quantitatively assessed (0, 0%; 1, < 10%; 2, 10-30%, 3, 30-60%, 4, 60-80% and 5, 80-100%). The following primary antibodies were used: anti-CCR4 (1:200, GTX21669, GeneTex, State of Texas, USA), anti-CXCR3 (1:500, GTX108145, GeneTex, State of Texas, USA), anti-P2RY14 (1:100, GTX71177, GeneTex, State of Texas, USA), anti-CCR2 (1:100, GTX21668, GeneTex, State of Texas, USA), anti-CCR8 (1:200, GTX100343, GeneTex, State of Texas, USA), and anti-CCL19 (1:100, GTX52595, GeneTex, State of Texas, USA). In the assessment of IHC staining, we employed a scoring system based on staining intensity and the percentage of positively labeled cells. Staining intensity ranged from 0 (negative) to 3 (high strength), while positive percentage scores spanned from 0 (no positive staining) to 4 (in more than 75% of cells). The final score resulted from multiplying these two scores. These scores were determined independently by two pathologists who were blinded to the patients' clinical features and outcomes.

### Statistical analysis

All data are expressed as mean ± standard deviation (SD). Statistical analyses were performed using GraphPad Prism 8.0. One-way analysis of variance and Tukey's test were used to compare differences between groups. Data homogeneity was tested using Bartlett's and Brown-Forsythe tests. Non-homologous data were analyzed using Brown-Forsythe ANOVA followed by Dunnett's T3 multiple comparison test.

## Results

### Stromal, immune, and ESTIMATE score patterns for different stages of HNSCC

**Figure 1:**
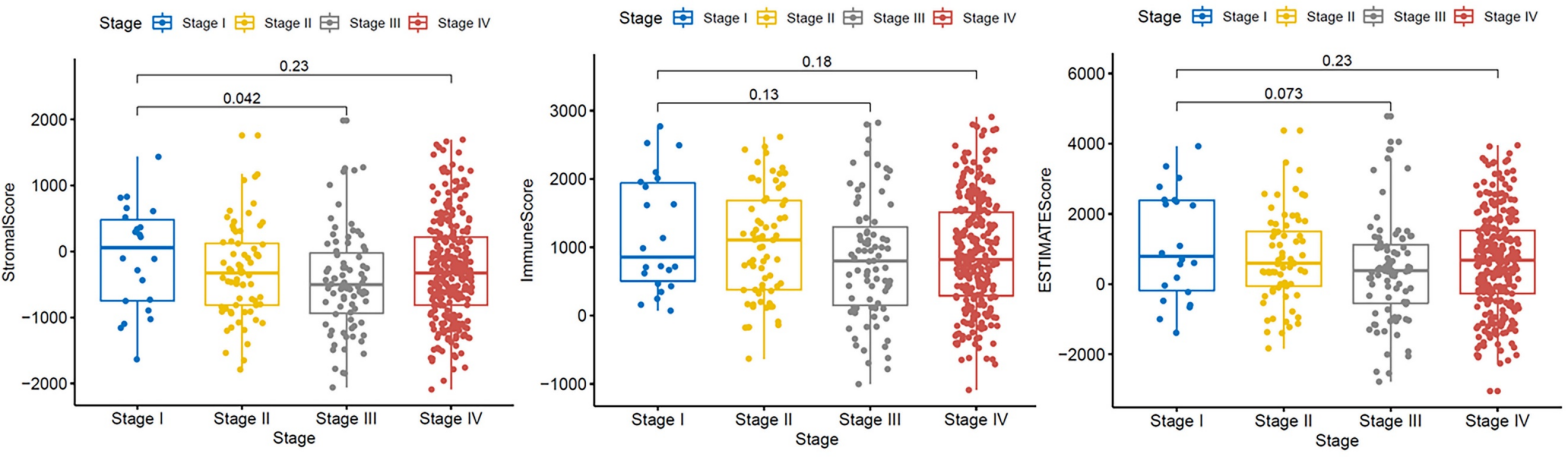
Correlation of immune and stromal scores with clinicopathological stage. Distribution of immune scores (P = 0.18), stromal scores (P = 0.23), and ESTIMATE scores (P = 0.23) for different tumor stages according to the Kruskal-Wallis rank-sum test.

A dataset covering different stages of HNSCC was obtained from the TCGA database to examine the distribution of stromal and immune scores and ESTIMATE scores across the different stages (Fig. 1). Our analysis revealed that the stromal and ESTIMATE scores of HNSCC were significantly different between patients with stage III and I HNSCC (P < 0.05). No other significant differences were observed according to the tumor stage. This result suggests that there were no significant differences in the expression of mesenchymal or immunoreactive genes between the different stages of HNSCC.

### Calculation of optimal cutoff scores and survival analysis

**Figure 2:**
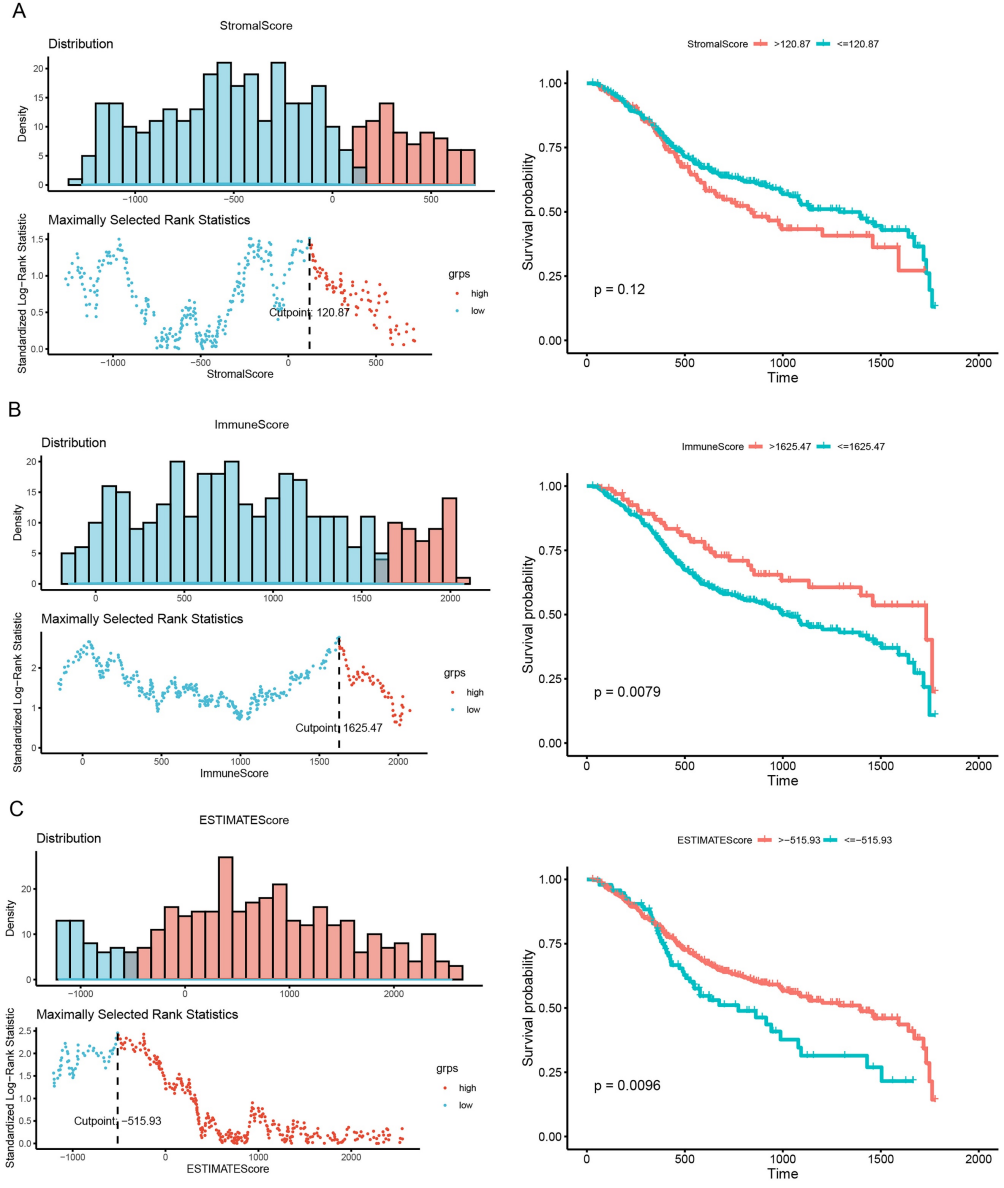
Correlation of scores with survival in patients with HNSCC. (A) Kaplan-Meier survival analysis of patients with HNSCC grouped into high- and low-score groups based on their stromal score (P = 0.0079, log-rank test). (B) Kaplan-Meier survival curve for high- and low-score groups according to immune score (P = 0.12, log-rank test). (C) Kaplan-Meier survival curves for patients with HNSC grouped into high- and low-score groups according to their ESTIMATE score (P = 0.0096, log-rank test).

As shown in Fig. 2, we calculated the optimal cutoff for the stromal score (Fig. 2A), immune score (Fig. 2B), and ESTIMATE score (Fig. 2C). Based on the optimal cutoff for the three scores, high- and low-score groups were created for survival analysis. The results of the survival analysis indicated that the immune and ESTIMATE scores were both significantly correlated with HNSCC prognosis (P = 0.0079 and P = 0.0096, respectively). This result implies that the abnormal expression of immune response genes may be associated with poor prognosis in HNSCC.

### Differential mRNA expression according to stromal score and immune score

**Figure 3:**
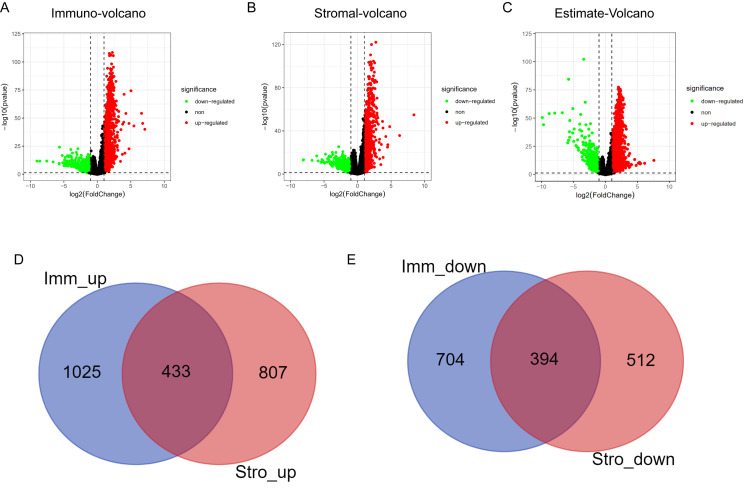
Volcano plots and Venn diagrams for DEGs. (A) Volcano plots for DEGs generated by comparison of the high score group with the low-score group based on the cutoff value for the immune score. DEGs were determined using the Wilcoxon rank-sum test (significance threshold: P = 0.05, fold-change > 1 after log2 transformation). (B) Volcano plots for DEGs based on the stromal score. (C) Volcano plots for DEGs based on the ESTIMATE score. (D, E) Venn diagrams showing the significantly upregulated and downregulated genes that were common across the immune and stromal score results.

Differential mRNA expression analysis was conducted on the high- and low-scoring groups based on the optimal cutoffs of stromal, immune, and ESTIMATE scores. The volcano plots in Fig. 3A-3C depicts the distribution of DEGs (threshold: |logFC| > 1, P < 0.01). The Venn algorithm was used to cross-validate the two groups of DEGs based on the immune and stromal scores. Altogether, 433 significantly upregulated and 394 significantly downregulated genes were identified (Fig. 3D, 3E). Therefore, we identified new genes related to immunity and matrix scores in the HNSCC microenvironment.

### Functional and pathway enrichment analysis of DEGs

**Figure 4:**
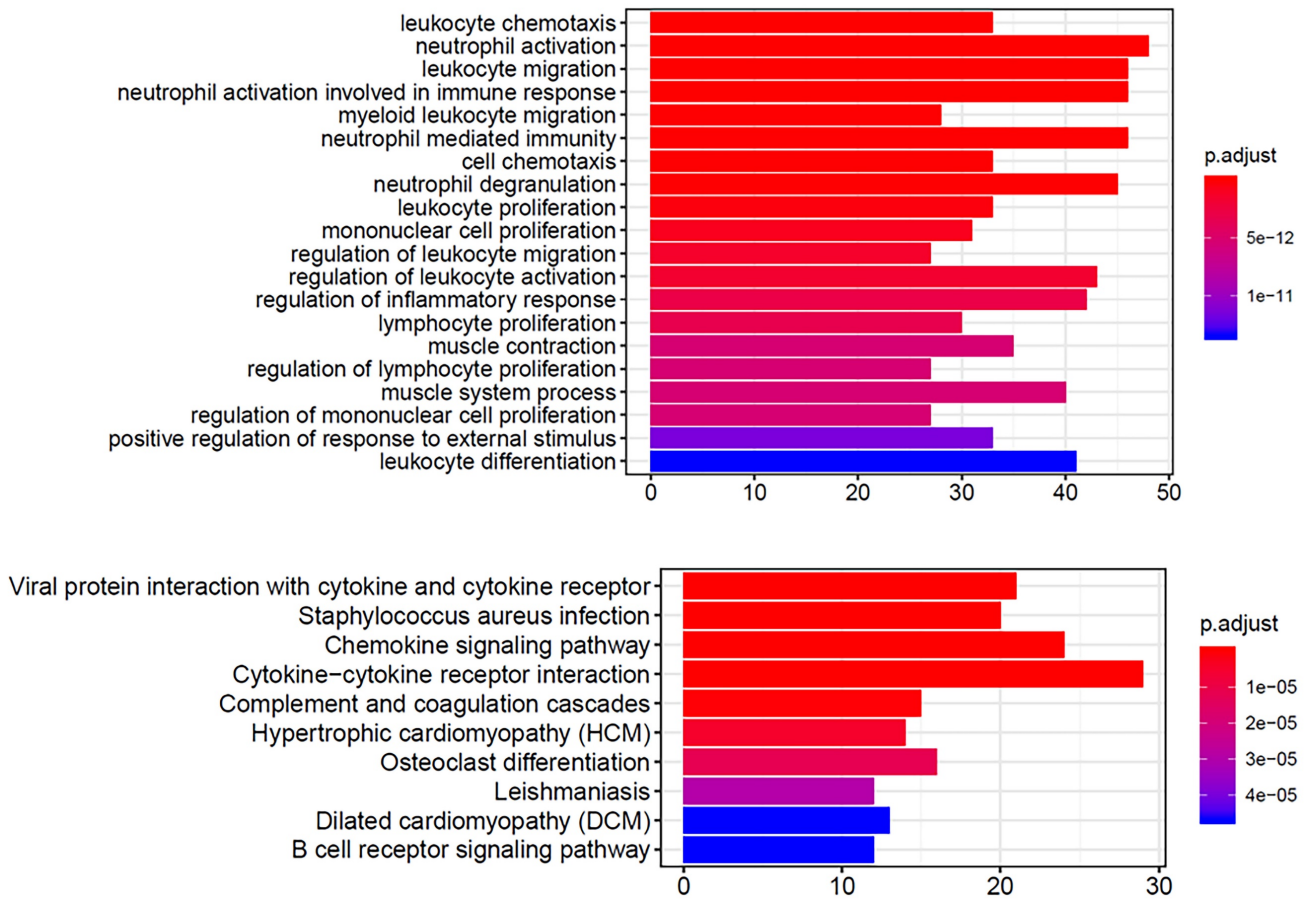
GO and KEGG enrichment analysis of DEGs In GO (A) and KEGG (B) enrichment analysis of 379 DEGs. Terms with P and q < 0.05 were considered to be enriched significantly. Significant enrichment of immune-related terms and pathways was observed.

We performed functional and pathway enrichment analyses on the significantly upregulated and downregulated genes using GO and KEGG analyses. The top 20 GO terms and top 10 KEGG pathways were analyzed. The GO terms were mainly related to immune cells (Fig. 4A), including neutrophils, leukocytes, and lymphocytes, and the KEGG results showed enrichment in cytokine-cytokine receptor interactions and chemokine signaling pathways (Fig. 4B). These gene function enrichment analyses synergistically indicated that the DEGs were closely associated with immune disorders in HNSCC, which may be the underlying mechanism for predicting HNSCC prognosis.

### PPI and ClueGO functional analysis of significantly upregulated genes

**Figure 5:**
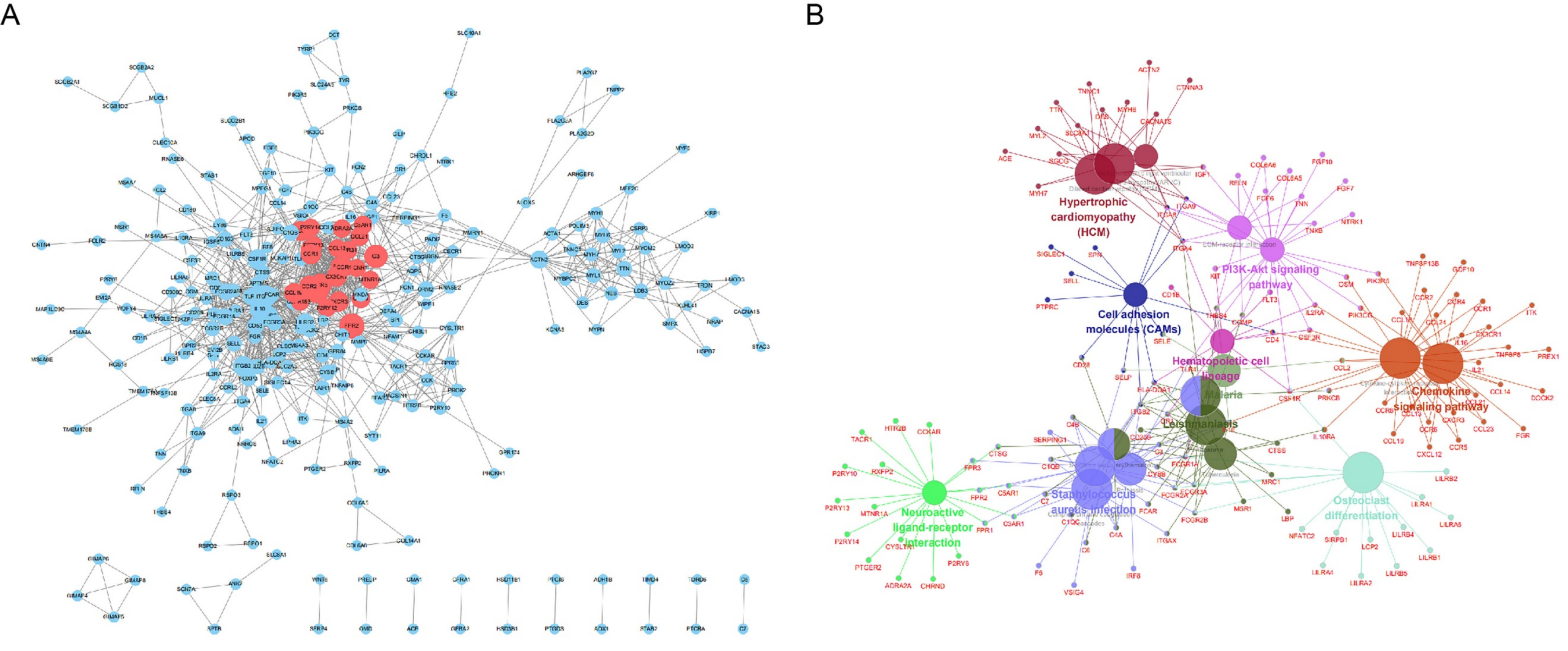
PPI analysis of 1,385 significantly upregulated genes in the STRING database Circles represent the degree of the corresponding node. The larger the circle, the greater the degree of the corresponding node and the higher the importance of the corresponding node in the network. (A) The red node represents the first key sub-network obtained using the MCODE plugin. (B) Functional analysis of upregulated genes using the ClueGO plugin for Cytoscape.

As shown in Fig. 5A, we constructed a PPI network for 1,385 significantly upregulated genes and analyzed 269 nodes and 1,140 pairs of interactions (protein interactions with a combined score > 0.7 were selected). Furthermore, the MCODE plugin was used to analyze the key sub-networks of the PPI network, and the first key sub-network was selected according to the threshold used in the analysis. We considered the 25 upregulated genes in the first sub-network as key genes for preliminary screening. The 25 upregulated genes were subjected to KEGG analysis using the ClueGO plugin (Fig. 5B). These results are consistent with those shown in Fig. 4.

### Survival analysis and multivariate regression analysis

**Figure 6:**
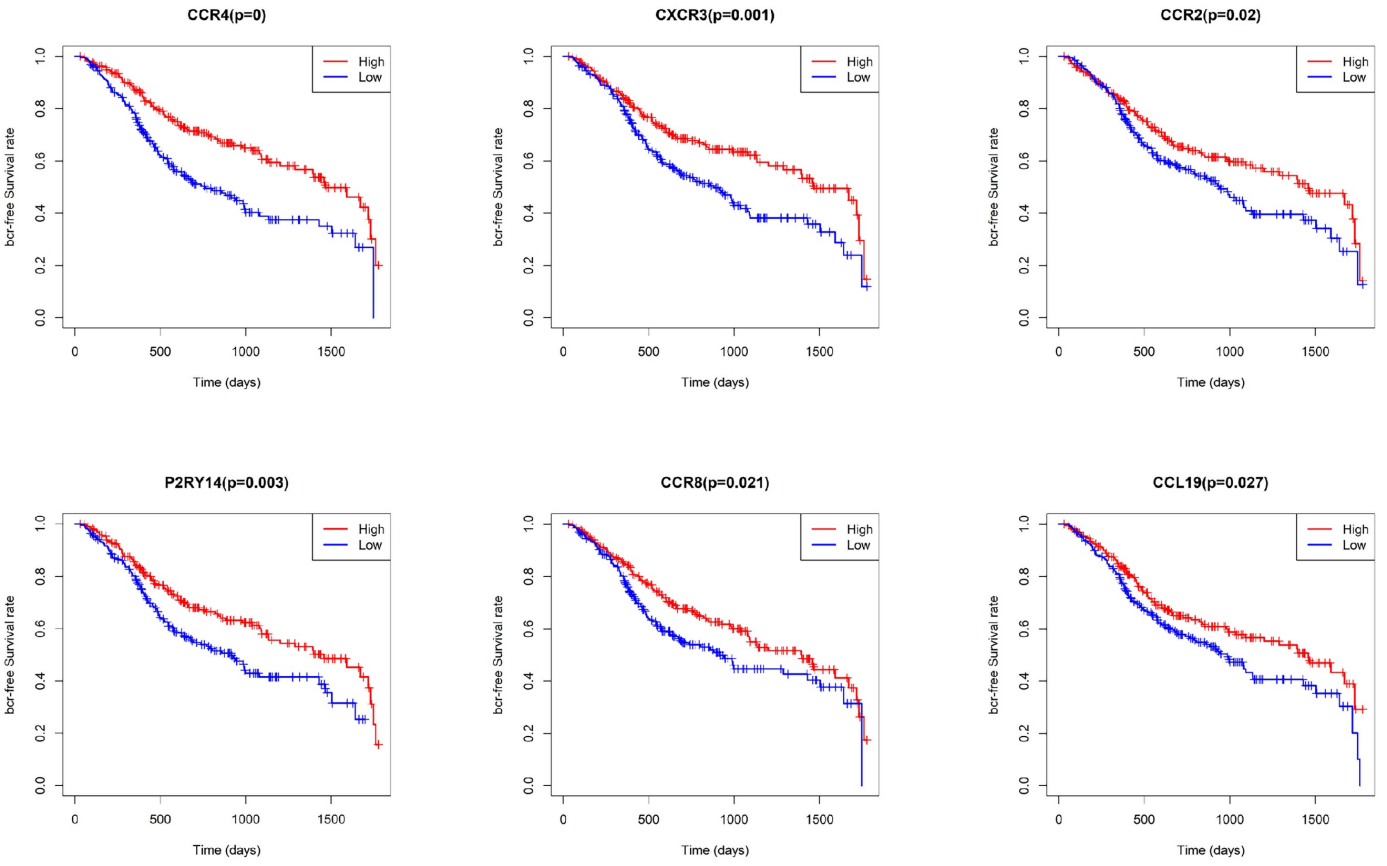
Survival analysis based on CCR4, CXCR3, P2RY14, CCR2, CCR8, and CCL19 expression. Significant differences in survival were found between cases with high and low expression of CCR4, CXCR3, P2RY14, CCR2, CCR8, and CCL19.

**Figure 7:**
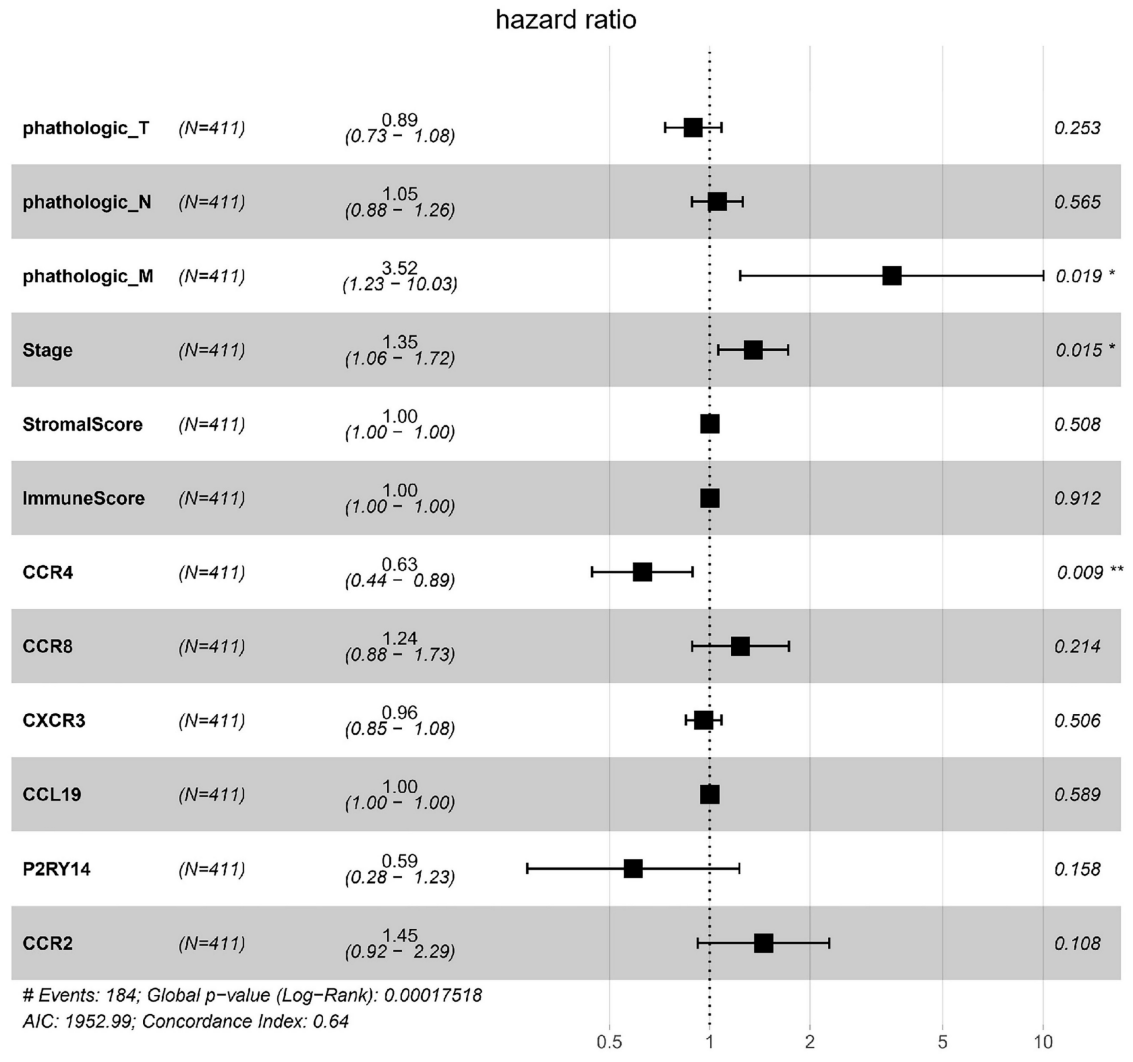
Multivariate regression analysis of expression of the significant genes, stromal score, immune score, T stage, N stage, M stage, and stage. Among the significant genes examined, CCR2 had the highest hazard ratio, while P2RY14 had the lowest hazard ratio. Left: protective factors; right: stimulating factors.

To identify key genes associated with HNSCC prognosis, we performed survival analysis on the 25 genes obtained from the preliminary screening described above. Among these, CCR4, CXCR3, P2RY14, CCR2, CCR8, and CCL19 were found to be significant in the survival analysis (Fig. 6). We then performed a multivariate regression analysis of the expression of these genes, stromal score, immune score, T stage, N stage, M stage, and clinical stage. Among the analyzed genes, CCR2 had the highest hazard ratio, whereas P2RY14 had the lowest (Fig. 7). Based on a bioinformatics statistical algorithm for tumor immunity and microarray sequencing, we explored a new target for HNSCC immunotherapy.

**Figure 8:**
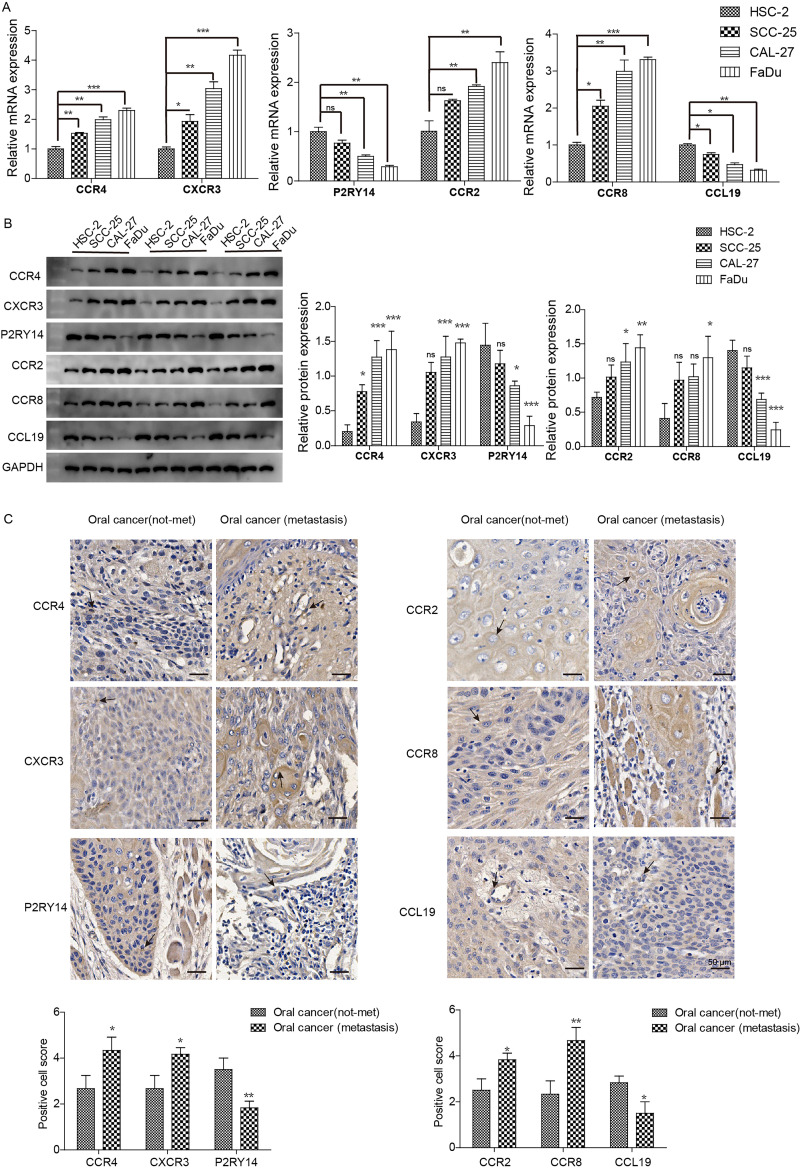
Expression of CCR4, CXCR3, P2RY14, CCR2, CCR8, and CCL19 in oral cancer tissues and highly invasive oral cancer cells. (A) mRNA expression was detected via qPCR. (B) Protein expression was detected via western blotting. Notable differences were observed in HSC-2 vs SCC-25 and CAL-27 vs FADU. (C) Immunohistochemical detection of gene expression levels in oral cancer tissues of patients with lymphatic metastasis and tissues of patients without tumor metastasis. Notable differences were observed in oral cancer (metastasis) vs. oral cancer (not-met). *p<0.05, **p<0.01, ***<0.001. “ns” means no correlation.

We further examined the expression of CCR4, CXCR3, P2RY14, CCR2, CCR8, and CCL19 in highly aggressive oral cancer cell lines and tissues. The expression of CCR4, CXCR3, CCR2, and CCR8 was higher in highly invasive oral cancer cell lines (SCC-25, CAL-27, and FaDu) than in the less aggressive cell line (HSC-2), whereas CCL19 and P2RY14 expression showed the opposite trend (Fig. 8A, 8B). In highly invasive FaDu cells, the difference in gene expression was the most significant, followed by the CAL-27 cell line. Only CCR4, CXCR3, CCR8, and CCL19 were differentially expressed in SCC-25 cells. Further analysis of gene expression in clinical tissues revealed that CCR4, CXCR3, CCR2, and CCR8 exhibited higher expression in oral cancer cells of patients with metastasis compared to those without, whereas CCL19 and P2RY14 displayed the opposite trend (Fig. 8C). Therefore, we identified CCR4, CXCR3, P2RY14, CCR2, CCR8, and CCL19 as potential targets for HNSCC immunotherapy.

## Discussion

In the present study, we analyzed gene expression data for HNSCC obtained from the TCGA database using a novel algorithm (ESTIMATE). This innovative approach allowed us to assess tumor purity and levels of stromal and immune cell infiltration. Our primary focus was to unveil the prognostic significance ingrained within the gene expression profiles of immune and stromal cells in tumors, identifying genes strongly linked to survival.

Because clinical HNSCC tissue samples contain cells other than tumor cells, gene expression analysis at the clinical level can be affected by the purity of the tumor cells 15. Therefore, our post-purity analysis of tumor and non-tumor cells in HNSCC clinical tissues provides a clearer picture of the gene expression profiles of HNSCC. Traditionally, tumor cell purity assessment relied on manual scrutiny of pathological staining maps, but this method had its limitations, notably subjectivity among observers 22,23. In contrast, our analytical methodology, as applied to HCSCC data in the TCGA database, overcomes these limitations. We achieved this by utilizing ESTIMATE, stroma, and immune scores to analyze tumor purity and variability in tumor gene expression. Survival analysis based on the immune and ESTIMATE scores showed that both scores were significantly associated with the prognosis of HNSCC, suggesting that abnormal expression of immune-related genes in HNSCC significantly affects prognosis.

Tumor, stromal, and immune cells have been observed to play a synergistic role in promoting HNSCC carcinogenesis and metastasis 24. Accordingly, in this study, we performed differential analysis of gene expression based on stromal and immune scores. In contrast, previous gene expression analysis studies have compared the gene expression profiles between tumor and paracancerous tissues 25. After analyzing the purity of the stromal and immune cells, we identified 433 significantly upregulated and 394 significantly downregulated genes. Thus, we identified key genes expressed in the TME. Furthermore, our investigation extended to PPI and GO analyses, confirming the enrichment of immune-related pathways within the identified DEGs.

Within the PPI network, we performed a critical sub-network analysis, identifying 25 genes in the first sub-network as key candidates for initial screening. These genes include ADRA2A, C3, C3AR1, C5AR1, CCL13, CCL19, CCL21, CCR1, CCR2, CCR4, CCR5, CCR8, CX3CR1, CXCL12, CXCR3, FPR1, FPR2, FPR3, GPR31, GPR183, MTNR1A, P2RY12, P2RY13, P2RY14, and SUCNR1. Notably, survival analysis singled out CCR4, CXCR3, P2RY14, CCR2, CCR8, and CCL19 as significant among these genes. According to the positions of amino acid residues, chemokines can be divided into four families: C, CC, CXC, and CX3C-chemokines 26. The CC chemokine receptor (CCR) family comprises receptors of the CC chemokine ligand. Similar to most chemokine families, the CCR family is primarily involved in the regulation of immune cell function 27. The CCR family plays an important role in cancer development through various anti-cancer and cancer-promoting functions 28. Furthermore, CCR4 can be used as a prognostic marker for immune infiltration in HNSCC 29. In malignancies, CXCR3 affects tumor progression by recruiting effector T cells 30. Furthermore, Meng et al. found that CCR4/8 and P2RY14 are associated with clinical stage and survival in patients with HNSCC. Moreover, CCR4/8 and P2RY14 may be involved in regulating the immune mechanisms of the TME, including regulating the cellular transport of various types of white blood cells. CCR4/8 and P2RY14 also drive Treg cell recruitment and participate in the regulation of stem cell compartments, thereby improving the prognosis of patients 31. The findings of our analysis are consistent with previous reports; however, they also provide a more accurate prediction of immune-related genes in HNSCC based on ESTIMATE. Specifically, these findings indicate that CCR4, CXCR3, CCR2, and CCR8 may affect the invasiveness of oral cancer cells. However, this result contradicts the expression trend of the results of the ESTIMATE analysis. We speculate that ESTIMATE analysis based on TCGA data may also have some limitations. TCGA data may have limitations due to potential inadequacies in sample representation across different infiltrating tissues. Additionally, the relatively small number of stage I tumor samples can restrict the scope of survival analysis and multiple regression analysis concerning immune-related genes based on infiltration levels. Furthermore, the ESTIMATE algorithm is currently in the exploratory stage. To analyze the data in more detail, the algorithm should be further optimized. In addition, the number of cell lines and clinical samples tested was limited, and further analysis with a larger sample size is required. Therefore, ESTIMATE analysis can only screen immune-related genes, but the specific roles of these genes need to be further explored in different infiltrating clinical samples and cells. The main innovation of our research revolves around the comprehensive analysis of stromal, immune, and ESTIMATE scores in HNSCC data from the TCGA database, ultimately culminating in the identification of key pathways, specifically the cytokine-cytokine receptor interaction and chemokine signaling pathway. These findings have been validated in highly invasive HNSCC tissues. Interestingly, we found that the expression of CCR4, CXCR3, CCR2, and CCR8 contradicted the bioinformatic analysis. However, it is undeniable that genes within this family hold significant importance in regulating the immune response in HNSCC, offering novel directions for future research.

In conclusion, the immune purity of TCGA-derived HNSCC tissues was determined using ESTIMATE. Tumor immune infiltration, tumor purity, survival, gene differential expression, protein-protein interaction, and functional enrichment analyses were evaluated based on matrix and immune cell scores. These results can be used as a basis for further research on the immune microenvironment of HNSCC. In the future, we envision the potential for precise characterization of the TME through scRNA-Seq analysis of HNSCC, aiming to pave the way for targeted prognostic therapies in patients with HNSCC.

## Figures and Tables

**Table 1:** Baseline characteristics of enrolled head and neck squamous cell carcinoma patients

	Metastasis patients	Non-metastatic patients
Sample number	10	10
Age	68.40±6.64	66.60±8.57
**Sex**		
Male	7(70%)	6(60%)
Female	3(30%)	4(40%)
Current smoker	5(50%)	6(60%)
Denture use	2(2%)	0
**T-stage**		
Ⅰ	0	3
Ⅱ	2	4
Ⅲ	5	1
Ⅳ	3	2

## Abbreviation

CCR: CC chemokine receptor
DEGs: Differentially expressed genes
ESTIMATE: Estimation of STromal and Immune cells in MAlignant Tumors using Expression data
FPKM: Fragments Per Kilobase of exon model per Million mapped fragments
GO: Gene ontology
HNSCC: Head and neck squamous cell carcinoma
KEGG: Kyoto Encyclopedia of Genes and Genomes
PPI: Protein-protein interaction networks
qRT-PCR: Real-time quantitative PCR
ssGSEA: Single-sample gene set enrichment analysis
TME: Tumor microenvironment
